# Surgical Operations in Private Practice

**Published:** 1904-10-29

**Authors:** John Aikman


### Hospital Clinics.

### SURGICAL OPERATIONS IN PRIVATE PRACTICE.

By John Aikman, M.D., C.M.Glasg.

Sir Lewis Morris is responsible for the division of a century's work into three generations of men: the grandfather, the father, and the son. Each of these generations, he says, marks a determinate progression, and is attended by varying conditions and unexpected developments. A riper knowledge raises the classes; it creates new industries for those who are young enough to learn; but it relentlessly oppresses those who cannot accustom themselves to the new conditions of life. Truly as this proposition may be applied to human affairs in general, its truth is more easily perceived when the records of various callings are separately examined, and nowhere is the process more conspicuous than in general medical practice. A fruit of modern advances in surgery is the growing inclination of general practitioners to undertake the performance of grave surgical procedures. The practice is one which offers possibilities of much good and great evil, according to the degree of skill and good judgment of the individual operator; and it is of some use to trace the phenomenon to its causes, which will be found in the great achievements of the last fifty years.

Three fairly distinct generations occupy the latter half of the 19th century; the grandfather is represented by the discovery of chloroform; the father took birth with the discovery of the antiseptic system; and the son with the new pathology of healing.

Each of these epochs sent the old-fashioned man into decay, and raised in his place a better operating surgeon and a better general practitioner. The use of chloroform extended the work and range of the surgeon, but it displaced the dashing operator whose handicraft and speed were his main claim to distinction. Lord Lister recognised this use of chloroform while the antiseptic system was germinating in his mind; for he pointed out to his class of surgery in the University of Glasgow in the session 1866–7 that Professor Chisholm, during the course of the Federal and Confederate war, had practised a system of dealing with gunshot wounds by a process which he called "remodelling," and which would have been impossible before the days of anæsthetics. It consisted of paring the edges of the ragged wound and bringing them together under conditions which favoured healing by first intention.

There was a throb of hope in the speech of the discoverer of antiseptic surgery that at some future day it might be possible to secure healing over substances which, like the bullet, were cleansed from the organisms which tend to produce suppuration in wounds. To-day that hope is realised in the indispensable buried suture. One further suggestion remains to the grandfather age; the late Sir James Paget in his "Surgical Pathology" described what he termed "healing by primary adhesion." He illustrated the process by the healing of razor-cuts and such specially clean-cut surfaces, but it was no more than prophetic of the healing by primary adhesion of serous membrane, which is so large a feature in modern surgery.

In the second generation, that of the father, we have the evolution of aseptic surgery by means of antiseptics. To this period belong the fierce struggles for a theory which it took years to substantiate, and the even more bitter controversies which ranged themselves round the names which indicated no more than cause and effect. Antiseptic treatment did not mean treatment by means of carbolic acid; it was a method which, properly understood, advanced the general practitioner into realms and responsibilities which had been heretofore the province of the surgeon. The pathologist took up the cause and worked hand in hand with the clinician, to the immense advantage of humanity.

There could be no finer picture of the third generation, that of the son, than is presented in Sir William MacEwen's Huxley address given a short while ago at Charing Cross Hospital. He deals with the triumphs of the second generation, and the successes and excesses of the third in the matter of operations on the appendix; and marks, as only the hand of a master can, the ethics of operations by general practitioners. He holds the balance between timidity and temerity.

The position of the general practitioner in regard to operations to be performed by him in private practice can be decided by a consideration of these three generations of surgeons, only with this reserve, that much must depend on the distance at which he resides from the best skilled aid. On outpost duty a man may face much. But every general practitioner should be a good anæsthetist, and he should have ingrained into his daily habit the methods by which asepsis is insured. He should be so keen and sensitive on this point as to check instantly the slightest fault or omission on the part of nurses or attendants, and he must be careful to provide a sufficient margin of sponges, instruments, and dressings to cover a large average of flaws. With so much a man may attempt what would have been forbidden to the grandfather.

A higher grade is reached by the man who has mastered the principles, first indicated by Paget, as to healing by primary adhesion, and has made himself an adept in dealing with the various tissues from skin to serous membranes; from the repair of a muscle or nerve-trunk to the repair of an intestine. Even such an one should not attempt the opening of the abdomen unless he has had ample experience as first assistant on similar occasions. Operations for the radical cure of hernia, and for appendicitis, unless urgent, fall under this criticism.

The general practitioner should be careful not unwisely to undertake exploratory incisions in the abdomen. If the alternative is death, he is responsible for having fitted himself to make a reasonably skilful effort; but the problems which an abdominal exploration opens up are often a tax upon the strongest head and the most fertile resource. Sir Frederick Treves says that in this department he would prefer a choreic hand to a faltering brain; the same is true of all operations which fairly fall to the general practitioner. No amount of manual dexterity, no amount of anatomical knowledge, no amount of self-confidence, will justify him in undertaking an operation which leaves room for *esprit des escaliers.*

